# Editorial: Animal biomechanics: application of the biomedical engineering to the veterinary sciences for the animal healthcare

**DOI:** 10.3389/fvets.2024.1390136

**Published:** 2024-03-25

**Authors:** Rocío Fernández-Parra, Alessia Di Giancamillo, Christian Peham, Mauro Malvè

**Affiliations:** ^1^Department of Veterinary Medicine and Surgery, Catholic University of Valencia (UCV) San Vicente Mártir, Valencia, Spain; ^2^Department of Biomedical Sciences for Health, University of Milan, Milan, Italy; ^3^Department of Companion Animals and Horses, University of Veterinary Medicine, Vienna, Austria; ^4^Department of Engineering, Public University of Navarra (UPNA), Pamplona, Spain; ^5^Biomedical Research Networking Center in Bioengineering, Biomaterials and Nanomedicine (CIBER-BBN), Madrid, Spain

**Keywords:** respiration physiology and drug delivery, animal orthopedics, animal kinematics, animal biomechanics, biomechanical test, medical devices and endoprosthesis, electrical impedance tomography, finite element analysis

Biomedical engineering is a discipline that combines engineering, biology and medicine. Within biomedical engineering, biomechanics uses the equations and laws of mechanics to solve problems and study phenomena relevant to the biomedical field. Traditionally, biomedical engineering has been used extensively in human medicine to improve human health and clinical outcomes. For example, it has been used to study pathologies and understand the function of cardiovascular and pulmonary mechanics, among other organs. It has enabled virtual surgical planning and the improvement of medical devices. More recently, considerable research has been devoted to mechanobiology, to study the motility and behavior of cells in different environments, with several applications in the study of human pathologies and tissue regeneration. Today, artificial intelligence is expected to drive future sophisticated applications.

The extensive research carried out over the last 30 years in this area has led to an increase in knowledge of a considerable number of aspects related to human health. With the increase in the power of computers and the parallel development of experimental set-ups, the complexity of numerical and experimental models that reproduce natural phenomena in human medicine and health care has increased considerably, allowing further improvements in related fields of research.

Surprisingly, biomedical engineering remains relatively underused in veterinary medicine. Despite its extensive applications in biology and human health, biomedical engineering has only occasionally been applied to veterinary medicine. However, the knowledge gained from previous applications of biomedical engineering in human medicine is readily available and suitable for direct implementation in veterinary medicine using similar technologies, protocols and workflows. For example, computational modeling based on patient-specific images can provide insights that are otherwise unattainable *in vivo* in both animals and humans. Artificial intelligence can analyze animal kinematics and stability to help diagnose disease. Experimental tests on animal tissue, originally intended for human health research, can be applied to animal health. In addition, three-dimensional (3D) additive printing for orthopedic purposes can facilitate the creation of customized endo- and exoprostheses uniquely tailored to the needs of animals.

This Research Topic therefore focuses on the application of biomedical engineering to veterinary science with the specific and precise aim of improving veterinary medicine and animal health. In this Research Topic, there is a collection of 14 papers that include some of the commented aspects and applications in different animals and techniques.

In the field of experimental animal orthopedics, Brabon et al. propose a Bayesian network model to study condylar fractures in horses. As these are commonly repaired with cortical screws, inadequate interfragmentary compression can lead to postoperative complications. Their investigation attempts to assess the relationships between several different fixation techniques and interfragmentary compression. They found moderate evidence that triangular repairs result in greater interfragmentary compression than linear repairs. Mendaza-DeCal et al. present and test a new 3D-printed endoprosthetic device with a specific protocol to mimic normal canine loading during walking, trotting and galloping. The research is a first step in the development of a valuable alternative to the well-known complex exo-endoprosthetic procedure for animals. Shi et al. examined the qualitative and quantitative morphological features of the porcine knee and anterior cruciate ligaments, comparing human and animal data reported in the literature. They found similar locations, orientations and basic morphology to humans, but different structure and dimensions. Lundin et al. develop a biomechanical test model of partial tarsal arthrodesis to determine whether a novel resorbable bone adhesive, phosphoserine-modified cement, provides measurable fixation strength in canine calcaneal arthrodesis without orthopedic implants. This work attempts to provide an alternative to arthrodesis. This invasive surgical procedure is used to treat canine and feline joints associated with a high incidence of complications. The results obtained in a cadaveric biomechanical test model showed that the resorbable adhesive can potentially contribute to the stability of arthrodesis surgery and can be evaluated as an alternative or complement to traditional fixation with metal implants.

Studies of large animal populations can help understand the effectiveness of pharmaceuticals. Huang conducts a comprehensive analysis of 131 metagenomic sequencing datasets from five species of non-human primates, including different regions and lifestyles, to analyze their resistance to antibiotics. As antibiotic resistance is a serious threat to animal health, the proposed comprehensive research will increase the knowledge of antibiotic management and disease prevention in a bidirectional way for both animal and human health.

Computational animal models using numerical algorithms and three-dimensional geometric discretisation have been widely used in human medicine, but few studies have focused on animal health. Xue et al. proposed a finite element analysis to study biomechanical changes in the rabbit knee joint. The computational results suggest that horizontal meniscal tears may not have a significant effect on the rabbit knee joint, and that different resection strategies may result in different biomechanical environmental changes.

The aim of this paper is to provide a reference for the future selection of experimental studies and clinical surgical strategies. Fernández-Parra et al. propose a computational fluid dynamics study focused on the treatment of asthma in cats. They evaluate the inhalation, deposition and transport of drug microparticles using discrete phase modeling and considering flows and particle sizes of salbutamol in a healthy cat model. The results suggest that therapy in animals differs from that in humans. In the same line, Zamora-Perarnau et al. investigated the effects of different airway management devices in cats under general anesthesia. They proposed a comparison of the performance of endotracheal tubes and supraglottic airway devices, showing that the correct size should be chosen carefully to avoid obstruction and reduce resistance. In their methods article, Burgos et al. introduce the in-house software Flowgy to the veterinary community. This software is a semi-automated tool designed to simulate human and animal airflow through the nasal passage to detect changes in flow. In this study, the authors tested the use and accuracy of Flowgy in lions with the aim of understanding the dynamics of nasal airflow in large felids for their health care and conservation.

Brabant et al. used non-invasive experimental methods for obtaining images of pulmonary ventilation in cattle to evaluate whether electrical impedance tomography variables could detect a difference in ventilation homogeneity between healthy and respiratory diseased cattle. Electrical impedance is a non-invasive imaging technique used to detect impedance changes where there is a change in the electrical conductivity of the body. Such changes are usually due to changes in the regional distribution of ventilation within the lung or changes in blood flow. The authors demonstrated that the proposed method, based on a belt with sensors connected to a computer, is a useful tool for the diagnosis of respiratory disease in cattle.

Animal kinematics and stability is also an important topic in this Research Topic. Non-invasive measurements can help in the early diagnosis of orthopedic diseases. Lutonsky et al. investigate the effect of external mechanical perturbations on stability in dogs using the body's center of pressure and demonstrate the effects of a balance training device in dogs. They show that the wobble amplitude of the platform controls the intensity of training programmes on motorized equipment. Virag et al. measured the center of pressure and ground reaction forces of 32 Labrador Retrievers and 17 Golden Retrievers at 4, 8, and 12 months of age to improve knowledge of canine hip dysplasia. The results showed significantly higher values for the center of pressure in the affected limb groups in both breeds at each measurement point during gait. The differences in measured parameters between limbs with healthy and diseased hip joints can be interpreted in terms of possible biomechanical adaptations and as an indicator of reduced stability. Mielke et al. apply semi-automatic deep learning digitization to a multivariate kinematic dataset in piglets to calculate joint angle profiles. The proposed workflow has potential for automated, accurate screening in livestock management. The overall aim of the study is to provide accurate diagnostic measurements, generated at high throughput, to improve animal welfare. Finally, Charalambous et al. investigate changes in vertical ground reaction forces, center of pressure and paw pressure in Belgian Malinois participating in obedience competitions during heelwork walking using a sensor-based experimental set-up. The measured variables are closely related and can be used to describe the effects of various factors such as aging, orthopedic, and neurological diseases.

In summary, the findings of the aforementioned papers underline a significant collection of pertinent applications of biomedical engineering in animal healthcare. However, they also indicate that numerous aspects remain ready for investigation concerning the intersection of these two disciplines.

## Author contributions

RF-P: Conceptualization, Writing—original draft, Writing—review & editing. AD: Writing—review & editing. CP: Writing—review & editing. MM: Conceptualization, Writing—original draft, Writing—review & editing.

